# Serum EV-Derived rno-let-7b-5p Is Associated with Lung–Adipose Alterations in Allergic Asthma

**DOI:** 10.3390/ijms27135989

**Published:** 2026-07-03

**Authors:** Wojciech Langwiński, Kosma Sakrajda, Zuzanna Stachowiak, Joanna Nowakowska-Lewicka, Maria Kachel, Emilia Cicha, Beata Narożna, Dawid Szczepankiewicz, Paweł Kołodziejski, Ewa Pruszyńska-Oszmałek, Aleksandra Szczepankiewicz

**Affiliations:** 1 Department of Experimental Biology, Poznan University of Medical Sciences, 8 Rokietnicka St., 60-806 Poznan, Poland; ksakrajda@ump.edu.pl (K.S.); alszczep@ump.edu.pl (A.S.); 2 Animal Facility, Poznan University of Medical Sciences, 8 Rokietnicka St., 60-806 Poznan, Poland; 3 Department of Animal Physiology and Biochemistry, Poznan University of Life Sciences, 60-637 Poznan, Poland; 4 Centre of Experimental Medicine, Poznan University of Medical Sciences, 8 Rokietnicka St., 60-806 Poznan, Poland

**Keywords:** allergic inflammation, obesity-associated asthma, miRNA, extracellular vesicles

## Abstract

Extracellular vesicles (EVs) and their miRNA cargo are important regulators of intercellular communication, but their role in connecting respiratory inflammation in asthma with systemic metabolic alterations remains unclear. We hypothesized that HDM-induced allergic inflammation alters selected serum EV-derived miRNAs and that these changes are associated with molecular alterations in lung and adipose tissue. Serum EV-miRNAs from control and HDM-induced allergic rats were isolated by size-exclusion chromatography and analyzed using TaqMan Advanced miRNA Assays. Candidate rno-let-7b-5p targets were integrated with lung and adipose tissue microarray data, validated by qPCR, and assessed in silico using IntaRNA and DMISO. Among the 13 analyzed miRNAs, rno-let-7b-5p showed a nominally significant 1.5-fold increase in serum EVs from allergic rats (unadjusted *p* = 0.03); no multiple-testing correction was applied. *Ptafr* and *Vav3* were enriched in lung inflammatory pathways, whereas *Zbtb16* was downregulated in adipose tissue. qPCR confirmed increased pulmonary *Ptafr* expression (*p* = 0.00802) and reduced adipose *Zbtb16* expression (*p* = 0.00034). Computational analyses yielded high-confidence predictions of interactions between rno-let-7b-5p and both genes, which require experimental validation. These preliminary, hypothesis-generating findings suggest that serum EV-associated rno-let-7b-5p may be associated with molecular alterations in the lung and adipose tissue during HDM-induced allergic inflammation. Confirmation in larger independent cohorts is required.

## 1. Introduction

Allergic asthma is a chronic inflammatory disorder of the airways characterized by airway hyperresponsiveness, mucus overproduction, and infiltration of eosinophils and T helper 2 (Th2) lymphocytes. The disease involves a complex interplay between immune cells, airway epithelium, and circulating mediators responsible for both local and systemic inflammation [1]. Existing studies suggest that asthma is not confined to the airways alone but also involves peripheral metabolic tissues, including adipose tissue [2].

Adipose tissue can modulate systemic inflammation through the production of various adipokines, either pro-inflammatory, such as leptin, or anti-inflammatory, such as adiponectin [3]. This may contribute to a state of chronic low-grade inflammation that potentially exacerbates airway inflammation. Obese patients with asthma respond poorly or develop resistance to drugs, such as bronchodilators and anti-inflammatory agents, as compared to non-obese asthmatics, thus contributing to the more frequent hospitalizations that make obesity-associated asthma an important global health problem [4].

Adipocytes have been shown to release extracellular vesicles (EVs), which are important mediators of intercellular communication. EVs can carry proteins and nucleic acids, such as microRNAs, from the parent cell to recipient cells. These adipose-derived miRNAs may exert systemic effects, including regulation of mRNA expression and translation [5]. Therefore, in inflammatory diseases such as allergic asthma, EV-mediated transfer of adipose-derived miRNAs may represent a potential mechanism linking adipose tissue inflammation with airway hyperresponsiveness and systemic immune dysregulation.

In our previous study, we demonstrated that upon allergic inflammation, both lung and adipose tissue showed altered miRNA expression profiles in a rat model of allergic asthma [6,7]. In this study, we aimed to compare the expression of these differentially expressed miRNAs in serum-derived extracellular vesicles to the expression previously observed in lung and adipose tissues. In addition, we sought to identify the potential target genes of these miRNAs and their expression profiles using multi-omic analysis and machine learning-based bioinformatic validation to accurately predict the most promising target gene interactions with EV-derived circulating miRNAs. We hypothesized that HDM-induced allergic inflammation alters the profile of serum EV-derived miRNAs and that these changes are associated with molecular alterations in lung and adipose tissue. The main research question of this study was whether serum EV-derived miRNAs may reflect a potential molecular link between respiratory inflammation and adipose tissue responses in allergic asthma.

## 2. Results

### 2.1. Confirmation of the Allergy Model

**Figure 1:**
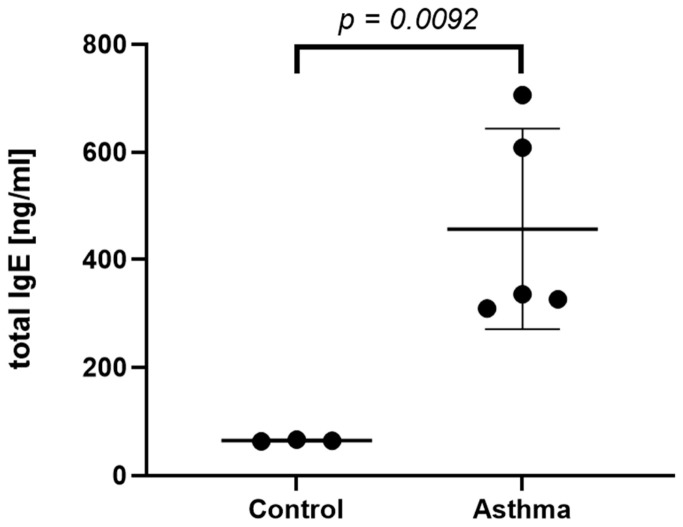
Total IgE concentration in serum from allergic rats (Asthma) compared to control animals (Control). Data were obtained using unpaired two-tailed Student’s *t*-test and are presented as a mean ± standard deviation (whiskers).

The presence of allergic inflammation was confirmed by measuring the total IgE concentration in serum, eosinophil count in peripheral blood and lung histology, as described previously [6,7]. In short, lung sections in allergized rats had distorted bronchiolar architecture and the accumulation of inflammatory cells in and around the bronchioles and alveoli. The analysis of peripheral blood revealed a marked increase in eosinophil count. We also found that total IgE concentration was approximately 7-fold higher in serum collected from rats exposed to HDM as compared to the control group (*p* = 0.009) (Figure 1).

### 2.2. Extracellular Vesicle Characterization

**Figure 2:**
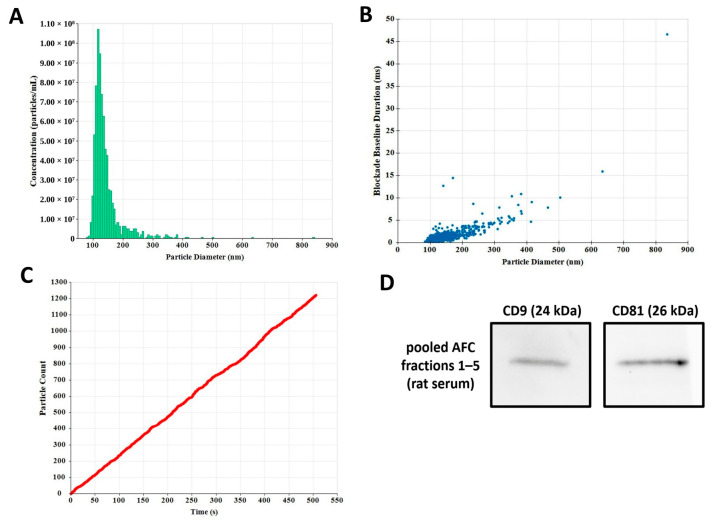
Extracellular vesicle characterization. (**A**) Size histogram of extracellular vesicles corresponding to particle concentration. (**B**) Blockade baseline duration (ms) for corresponding particle diameter (nm). (**C**) Particle measurement rate over time during the measurement. (**D**) Detection of EV membrane markers with Western blot.

For EV characterization, we used TRPS analysis with a qNano Gold instrument (Izon). The raw particle concentration measured was 7.66 × 10^8^ particles/mL, with an average particle rate of 144.9 particles/min and a total particle count of 1223. The mean and mode particle diameters were 142 nm (Std. Dev. = 52.2) and 117 ± 3.0 nm, respectively (Figure 2A,B). As shown in Figure 2C, the particle count remained constant throughout the measurement. As shown in Figure 2D, the tetraspanin EV-specific membrane markers CD9 and CD81 were detected with Western blot in pooled fractions 1–5. Full Western blot membranes for CD81 and CD9 are shown in Figures S1 and S2, respectively.

### 2.3. Quantitative RT-PCR for miRNA

**Figure 3:**
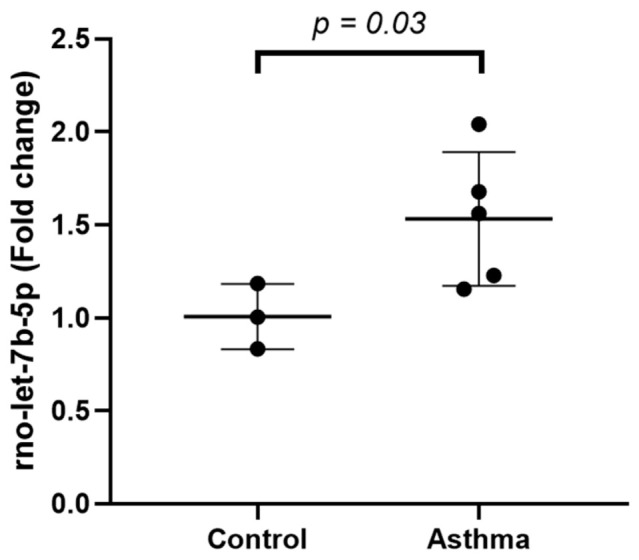
Fold change in rno-let-7b-5p expression in serum-derived EVs from allergic rats compared to control animals. Expression is normalized to endogenous control rno-miR-26a. Data were obtained using unpaired two-tailed Student’s *t*-test and are presented as scatter plots with mean and standard deviation as whiskers.

To quantify the expression of miRNAs in serum-derived EV, we performed real-time PCR with TaqMan probes. We focused on miRNAs that demonstrated significantly altered expression in our previous experiments in lungs and adipose tissues. Among the 13 miRNAs analyzed, rno-let-7b-5p showed a nominally significant 1.5-fold increase in serum EVs from allergic rats compared with control animals (unadjusted *p* = 0.03). Because no multiple-testing correction was applied, this result should be interpreted as exploratory and hypothesis-generating (Figure 3).

### 2.4. Microarray Gene Expression

**Figure 4:**
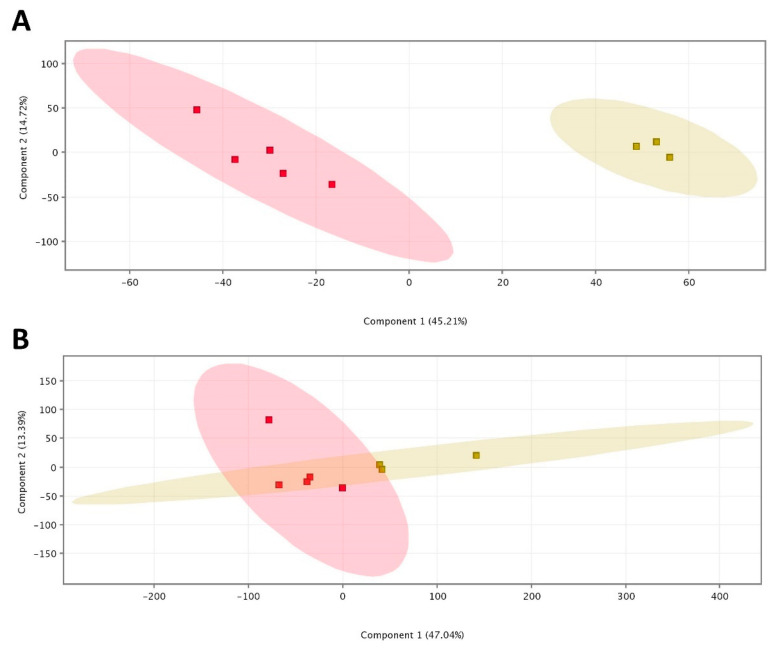
PCA for lung (**A**) and adipose tissue (**B**) from asthmatic (red) and control (yellow) rats. The first two principal components jointly explained 59.93% of the variance in lung tissue and 60.43% in adipose tissue.

For gene expression analysis with microarrays, we used GeneSpring 15.5 GX. A total of 45,738 rat gene probes were analyzed for both lung and adipose tissues. After applying a fold change threshold >1.5, 4330 genes for the lungs and 11,129 genes for the adipose tissue remained. The first and second principal components explained 59.93% and 60.43% of the variance in lung and adipose tissue, respectively. The principal component analysis (PCA) for asthmatic (red) and control (yellow) rats in lung and adipose tissues is shown in Figure 4A and Figure 4B, respectively.

**Figure 5:**
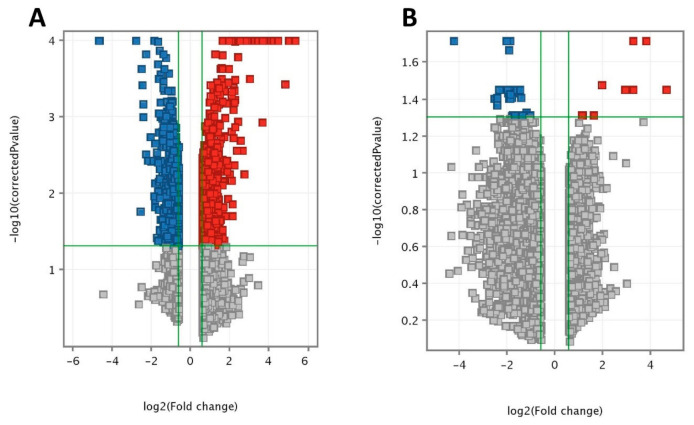
Volcano plot of downregulated (**left**) and upregulated genes (**right**) in the lungs (**A**) and adipose tissue (**B**). The fold-change threshold was set at 1.5.

In the lungs, 1319 genes were differentially expressed, with 776 genes upregulated and 543 downregulated (Figure 5A). In the adipose tissue, 48 genes were differentially expressed, with nine genes upregulated and 39 downregulated (Figure 5B). The lists of differentially expressed genes for the lungs and adipose tissue are presented in Supplementary Files S1 and S2, respectively.

### 2.5. Multi-Omic Analysis (MOA)

**Figure 6:**
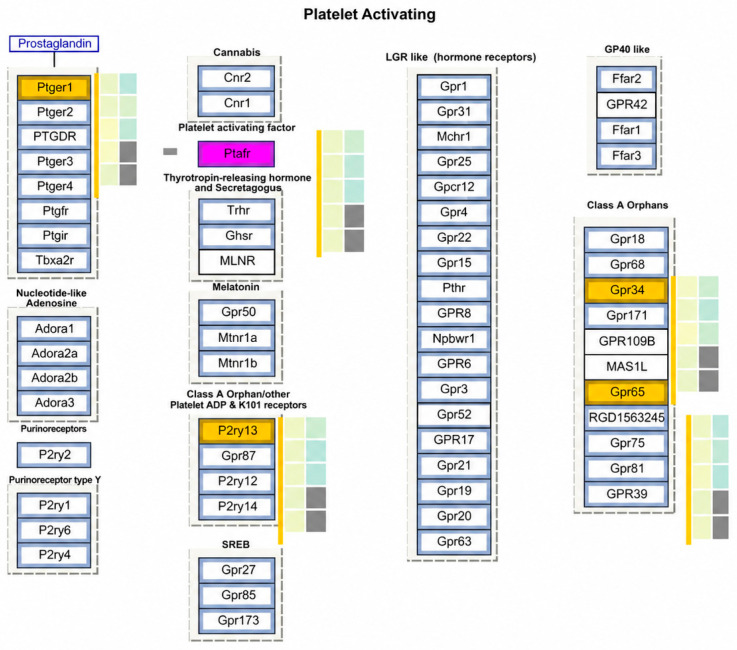
*Ptafr*-associated pathway 1—“GPCR Class A Rhodopsin-like”. Genes significantly altered in the expression microarray experiment and identified by prediction databases as target genes for rno-let-7b-5p were indicated with yellow and blue gene boxes, respectively. Genes (*Vav3* or *Ptafr*) enriched in both datasets were indicated with a pink gene box.

**Figure 7:**
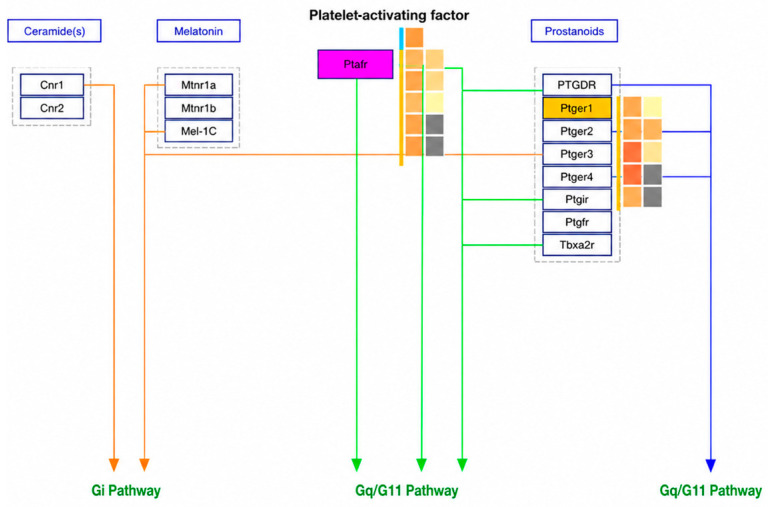
*Ptafr*-associated pathway 2—”Small Ligand GPCRs”. Genes significantly altered in the expression microarray experiment and identified as target genes for rno-let-7b-5p were indicated with yellow and blue gene boxes, respectively. Genes (*Vav3* or *Ptafr*) enriched in both datasets were indicated with a pink gene box.

**Figure 8:**
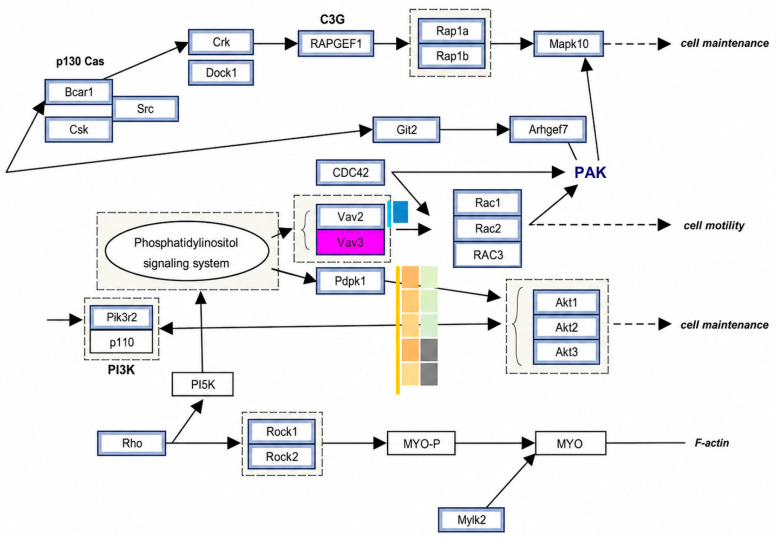
*Vav3*-associated pathway 1—”Integrin-mediated cell adhesion”. Genes significantly altered in the expression microarray experiment and identified as target genes for rno-let-7b-5p were indicated with yellow and blue gene boxes, respectively. Genes (*Vav3* or *Ptafr*) enriched in both datasets were indicated with a pink gene box.

**Figure 9:**
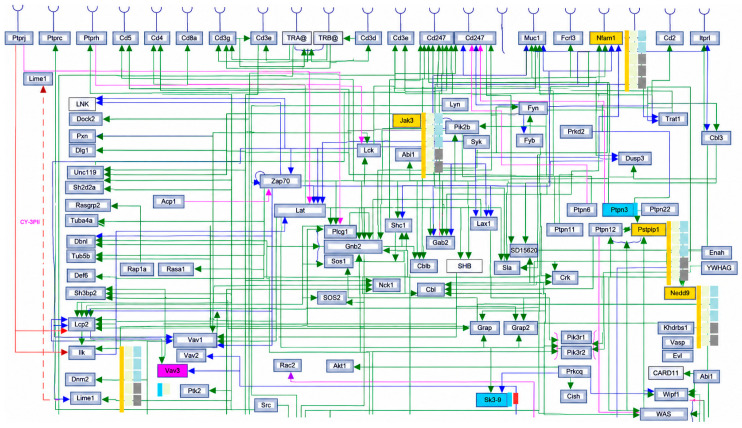
*Vav3*-associated pathway 2—”T-cell receptor signaling pathway”. Genes significantly altered in the expression microarray experiment and identified as target genes for rno-let-7b-5p were indicated with yellow and blue gene boxes, respectively. Genes (*Vav3* or *Ptafr*) enriched in both datasets were indicated with a pink gene box.

A multi-omic analysis module in GeneSpring 15.5 GX was used to associate rno-let-7b-5p targets with significantly altered genes identified in expression microarrays. In lung tissue, we found that *Ptafr* (Platelet-activating factor receptor) and *Vav3* were significantly enriched in four pathways. *Ptafr* was associated with “GPCR Class A Rhodopsin-like” (Figure 6) and “Small Ligand GPCRs” (Figure 7) pathways, whereas *Vav3* was enriched in “Integrin-mediated cell adhesion” (Figure 8) and “T-cell receptor signaling pathway” (Figure 9). Although no significantly enriched biological pathways were identified in adipose tissue, we found that *Zbtb16* showed significantly decreased expression in this tissue and was identified as an experimentally validated target gene of rno-let-7b-5p. The cropped pathway maps highlighting the overlapping genes of interest are presented in Figure 6, Figure 7, Figure 8 and Figure 9, whereas the complete pathway maps generated using GeneSpring are provided in the corresponding Supplementary Figures S3–S6.

### 2.6. Validation of Gene Expression

**Figure 10:**
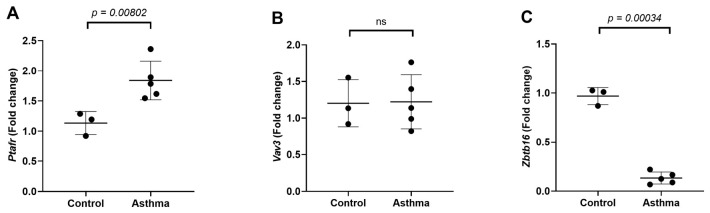
Fold change in gene expression for *Ptafr* (**A**), *Vav3* (**B**) in lungs, and *Zbtb16* (**C**). Expression was analyzed in lungs and adipose tissue from allergic rats as compared to control animals. Data were obtained using unpaired two-tailed Student’s *t*-test and are presented as scatter plots with mean and standard deviation as whiskers.

Using qPCR, we further analyzed the gene expression of *Vav3* and *Ptafr* in the lungs of allergic rats as compared to control animals. Additionally, we also validated the expression of *Zbtb16* in adipose tissues from allergic rats as compared to control animals. We found significantly increased expression of *Ptafr* (*p* = 0.00802) (Figure 10A) in lungs and decreased expression of *Zbtb16* (*p* = 0.00034) (Figure 10C) in adipose tissue. No significant differences in gene expression were found for *Vav3* (Figure 10B).

### 2.7. miRNA–RNA Interaction Analysis

**Figure 11:**
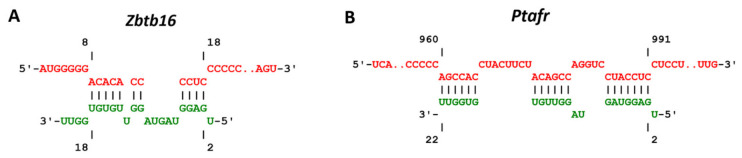
Graphical representation of the interactions between *Zbtb16* (**A**), *Ptafr* (**B**) and rno-let-7b-5p. Different colors represent the sequence of target genes (red) and rno-let-7b-5p (green).

**Figure 12:**
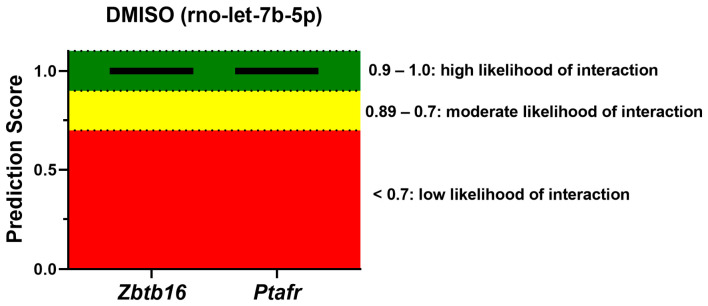
Prediction score for *Ptafr*, *Zbtb16*, and rno-let-7b-5p binding.

Interaction analysis was performed only for *Ptafr* and *Zbtb16*, as only these genes showed significantly altered expression in the real-time PCR validation in lung and adipose tissue. The predicted interaction energy scores for *Zbtb16* and *Ptafr* were −8.44 kcal/mol and −17.87 kcal/mol, respectively, suggesting spontaneous formation of miRNA–mRNA duplexes within the UTR regions of the target genes. A graphical representation of the interactions between rno-let-7b-5p and its target genes is shown in Figure 11A,B. In a machine learning-based analysis of miRNA–target binding, DMISO confirmed strong interactions between rno-let-7b-5p and its target genes. The prediction scores were 0.9987 for *Zbtb16* and 0.9997 for *Ptafr* (Figure 12), indicating high-confidence interactions. These high scores suggest a high-confidence computational prediction, as values close to one represent very high interaction probability, while values in the range of 0.7–0.9 are interpreted as a moderate likelihood of interaction. However, this computational prediction requires experimental validation.

## 3. Discussion

In this preliminary study, serum EV-associated rno-let-7b-5p showed increased expression in HDM-exposed rats compared with control animals. This finding was accompanied by previously observed decreased rno-let-7b-5p expression in lung tissue and altered expression of its predicted targets, including increased pulmonary *Ptafr* and decreased adipose Zbtb16. These observations indicate associations across serum EVs, lung tissue, and adipose tissue but do not establish a causal inter-organ mechanism. We previously demonstrated a decreased expression of rno-let-7b-5p in lung tissue [6], and the level of *Ptafr*, a target of this miRNA, was increased in asthmatic rats compared to control animals. In turn, the expression of *Zbtb16* was significantly decreased in adipose tissue from asthmatic rats compared to control animals, which might be a consequence of the uptake of EV-derived rno-let-7b-5p by adipose tissue. Furthermore, multi-omic analysis confirmed significant pathway enrichment associated with *Ptafr* in the lungs, whereas no significant pathways were identified for *Zbtb16*.

The link between allergic asthma and adipose tissue has been postulated; however, despite numerous studies, the exact pathomolecular mechanism has not yet been elucidated [8]. It has been suggested that circulating cytokines (IL-6, IL-10, TNF-α, IL-1β, and MCP-1) and adipokines (leptin and adiponectin) secreted by white adipose tissue (WAT) may induce low-grade chronic inflammation, which could contribute to the development of allergic asthma. For instance, increased release of TNF-α by WAT enhances the activity of T-helper type 2 (Th2) lymphocytes, leading to the secretion of allergic cytokines such as IL-4 and IL-5 by bronchial epithelial cells. Additionally, increased concentrations of circulating IL-6 and leptin, along with the downregulation of IL-10, impair the regulatory activity of Treg cells, which in turn significantly reduces immunological tolerance to antigens and fosters allergen sensitization [9].

In turn, cytokines (TNF-α, IL-1β, and IL-6) produced by the lungs during allergen sensitization and released into the bloodstream are taken up by adipose tissue, where they induce adipocyte inflammation and hypertrophy, which in turn may contribute to obesity [10]. We have previously shown that HDM-induced allergic eosinophilic inflammation in the rat lungs leads to a significant increase in total body fat, blood glucose concentration, and body weight [7]. At the molecular level, allergic lung inflammation significantly increased the gene expression of leptin, insulin receptor, Tnf-α, and Il-6 in adipose tissue from asthmatic rats as compared to control animals.

Recent studies increasingly support the role of EV-associated miRNAs in asthma pathogenesis. Altered EV-miRNA expression profiles have been reported in bronchoalveolar lavage fluid, sputum and plasma from asthmatic patients compared with healthy controls [11]. For example, levels of has-miR-92b, has-miR-210, and has-miR-34a were found to be dysregulated during asthma development and correlated with lung function parameters, highlighting the relevance of EV-mediated communication in airway disease [12]. Similarly, Soccio et al. demonstrated that exosomal miRNA profiles differ according to asthma severity and identified has-let-7a-5p, has-miR-21-5p and miR-223-5p as asthma-associated EV-miRNAs [13]. Likewise, Bahmer et al. identified hsa-miR-122-5p as a candidate asthma-associated EV-miRNA based on differential expression and bioinformatic analyses, highlighting the utility of circulating EV-miRNAs for identifying molecular mechanisms and potential biomarkers of asthma [14]. Furthermore, Alhamdan et al. reported that plasma EV-miRNA signatures in obesity-associated asthma (OA) were linked to both inflammatory and metabolic pathways, supporting the concept that circulating EV-miRNAs may reflect communication between respiratory and peripheral metabolic tissues [15]. In particular, the miR-17–92 and miR-106a–363 clusters were differentially expressed in OA patients compared with healthy controls. Collectively, these findings support the growing evidence that EV-derived miRNAs participate in asthma pathogenesis and may provide insight into both local airway inflammation and systemic disease-associated processes.

One possible mechanism contributing to the pathogenesis of asthma may involve the transmission of inflammatory stimuli from adipose tissue via microRNAs carried as cargo within EVs secreted into the bloodstream. In this study, we analyzed the expression of EV-derived miRNAs that showed significantly altered changes in the lungs and adipose tissue in a rat model of asthma. Among all analyzed genes, only rno-let-7b-5p showed significant upregulation in EVs isolated from the serum of asthmatic rats compared with control animals. Conversely, the expression of this miRNA was markedly decreased in the lung tissue of allergic rats, which may reflect its increased release into circulation relative to controls.

The available information on the expression of miR-let-7b-5p in the pathogenesis of asthma remains limited. Previous studies demonstrated that miR-let-7b expression is decreased in airway epithelial cells from individuals with both mild and severe asthma, compared to healthy controls [16]. However, the potential involvement of EV-associated let-7b-5p in coordinated molecular responses between the lung and adipose tissue during allergic inflammation remains poorly characterized. Despite that, miRNAs from the let-7 family (including let-7b) were postulated to act as negative regulators of inflammatory genes such as NF-κB, IL-6, IL-10, IL-12, IL-17, and GM-CSF [16,17].

Interestingly, the release of let-7b-5p as cargo of EVs secreted by human airway epithelial cells (AECs) was previously described by Koeppen et al. [18] and it was shown to be responsible for reducing biofilm formation and increasing the antibiotic sensitivity of *Pseudomonas aeruginosa*. In another study by Hartung et al. [19], EV-derived expression of let-7b-5p was significantly increased in the sputum of patients with nonsteroidal anti-inflammatory drug-exacerbated respiratory disease (N-ERD) compared to healthy controls. The latter is a chronic type 2 inflammatory disease of the upper and lower airways, associated with asthma and characterized by severe eosinophilia. These results are in line with our observation of increased expression of serum EV-derived rno-let-7b-5p, potentially released from inflamed lung tissue directly into the circulation. Although let-7b-5p has previously been implicated in asthma and identified as a component of extracellular vesicle cargo, the present study extends these observations through an integrated cross-tissue approach. Specifically, serum EV-derived miRNA expression was combined with matched transcriptomic data from lung and adipose tissue obtained from the same HDM-exposed animals. This design enabled the identification of tissue-specific associations between circulating rno-let-7b-5p and molecular alterations involving *Ptafr* in the lung and *Zbtb16* in adipose tissue. Therefore, the novelty of the present study lies in the integration of circulating EV-miRNA profiles with respiratory and metabolic tissue responses in a defined experimental model, rather than in the identification of let-7b-5p itself.

Additionally, in our study, we validated the expression of *Ptafr* and *Zbtb16*, predicted targets of rno-let-7b-5p. Furthermore, using IntaRNA, we calculated the binding energy and steric interactions between rno-let-7b-5p and its target genes, which were further validated with the machine learning-based tool DMISO.

Data on the interaction between PTAFR and let-7b-5p are limited. Rattanapan et al. [20] reported that PTAFR was predicted to be a target gene for let-7b-5p and involved in the pathogenesis of diabetes mellitus. The authors suggested that increased PTAFR expression might enhance platelet reactivity and inflammation by increasing platelet sensitivity to PAF. This process could be mediated by elevated oxidative stress, NADPH oxidase activity, and increased production of reactive oxygen species (ROS).

*Ptafr* encodes the platelet-activating factor receptor, a G protein-coupled receptor (GPCR) within the Class A/1 (Rhodopsin-like) family. In our multi-omic analysis, Ptafr was significantly enriched in the “GPCR Class A Rhodopsin-like” pathway, consistent with its role as a key component of this membrane receptor class responsible for binding platelet-activating factor (PAF). This binding triggers several downstream signaling cascades, including MAPK/ERK, PI3K, JAK2, and NF-κB, which regulate critical biological processes such as cell migration, proliferation, and inflammation [21,22]. The increased expression of PTAFR, resulting from the decreased level of rno-let-7b-5p due to its potential release into the circulation, leads to enhanced uptake of PAF and upregulation of IL-1β, IL-6, and TNF-α, which play crucial roles in the pathogenesis of asthma. Furthermore, increased PAF uptake activates the nucleotide-binding domain leucine-rich repeat-containing protein 3 (NLRP3) inflammasome that processes precursors of IL-18 and IL-1β into their active, biologically functional forms, resulting in their increased activity [23]. Interestingly, NLRP3 and IL-18 were often associated with severe asthma, contributing to chronic Th2- and non-Th2-dependent airway inflammation, airway hyperresponsiveness, and mucus overproduction [24]. As an example, in a study by Hur et al. [25], obese mice fed a high-fat diet (HFD) and sensitized with ovalbumin (OVA) were treated with a glucagon-like peptide-1 receptor (GLP-1R) agonist and an anti-obesity drug. Treatment with the GLP-1R agonist not only reduced body weight but also suppressed eosinophilic airway inflammation and airway hyperresponsiveness (AHR). Surprisingly, inflammatory lung changes in the obesity-associated asthma mice model were driven by the NLRP3 inflammasome and IL-1β, both of which were further reduced after GLP-1R agonist treatment.

Additionally, in a second pathway identified in our multi-omic analysis, *Ptafr* was also associated with platelet activation, positioned upstream of the Gq/G11 signaling pathway. This leads to the activation of phospholipase C-beta (PLC-β), further contributing to intracellular signaling events, such as the hydrolysis of membrane lipid phosphatidylinositol 4,5-bisphosphate (PIP2), which produces inositol 1,4,5-trisphosphate (IP3) and diacylglycerol (DAG) [26]. A downstream consequence of this event is the release of Ca^2+^ from intracellular stores in the endoplasmic reticulum (ER) into the cytosol and the activation of protein kinase C (PKC) [27]. This, in turn, induces further platelet-activating factor (PAF) formation and the production of secondary lipid messengers such as leukotrienes and prostaglandins, which are released during the late-phase response, inducing airway inflammation and remodeling, associated with obesity-associated asthma [28,29].

The second analyzed target for rno-let-7b-5p is *Zbtb16* (Zinc finger and BTB domain-containing 16), also known as *Plzf* (promyelocytic leukemia zinc-finger), which belongs to the zinc finger superfamily of multifunctional transcription factors involved in gene regulation, cell differentiation, and embryonic development [30]. It was shown that *Zbtb16* interacts in the nucleus with ASC (apoptosis-associated speck-like protein containing a CARD), which is a crucial component for inflammasome oligomerization and assembly [31,32]. Dong et al. [33] showed that *Zbtb16* promotes the conjugation of the SUMO (Small Ubiquitin-like Modifier) protein to ASC, thereby facilitating inflammasome assembly and the production of pro-inflammatory cytokines such as IL-1β and IL-18. In another study, Pobezinsky et al. [34] confirmed that the let-7 miRNA family targets *Zbtb16*, which encodes the PLZF transcription factor, crucial for determining NKT cell differentiation. The authors demonstrated that upregulation of let-7 miRNAs during NKT thymocyte differentiation hampers PLZF activity, resulting in the generation of interferon-γ-producing NKT1 cells. In contrast, downregulation of the let-7 miRNA family promotes differentiation into IL-4-producing NKT2 and IL-17-producing NKT17 cells. In the presence of asthma and lung-secreted allergic cytokines (such as TNF-α, IL-1β, and IL-6) that enter the circulation and reach adipose tissue, inflammation can be induced locally that will result in adipocyte hypertrophy. We hypothesize that this might lead to increased expression and uptake of rno-let-7b-5p by adipose tissue. This, in turn, could significantly reduce *Zbtb16* expression, impair inflammasome assembly, and promote the differentiation of IL-17-producing NKT17 cells in the thymus. It is noteworthy that IL-17 is a key cytokine known to inhibit adipogenesis, modulate adipose tissue expansion, and regulate glucose metabolism [35]. This mechanism may help to limit chronic low-grade inflammation in adipose tissue, but further functional studies are necessary to verify this hypothesis.

A major strength of our study lies in the integration of data collected from multiple tissues (lungs and adipose tissue) and its correlation with findings from EV-derived miRNAs. Furthermore, the use of next-generation sequencing (NGS), expression microarrays, and machine learning-based tools significantly enhanced the accuracy of miRNA target prediction.

Future studies should, however, identify the exact cellular origin of serum EV-associated rno-let-7b-5p to determine whether its altered circulating level reflects release from the inflamed lung, adipose tissue, immune cells, or other cell populations. Such analysis is, however, challenging, as it would require lung tissue dissociation, cell sorting, and the analysis of individual EVs at the nanomolar level. Additionally, under stress conditions (such as shear forces or elevated temperatures), cells can significantly increase EV release, which could potentially skew the obtained results [36]. Additionally, the sample size was relatively small, particularly in the control group, which limits statistical power.

Finally, only one HDM-induced allergic asthma protocol with male rats and one experimental time point were analyzed. Thus, the present study does not address whether EV-derived miRNA expression changes dynamically during sensitization, challenge, or resolution of allergic inflammation. Finally, the lack of direct functional validation is a limitation of the present study. However, gain- and loss-of-function experiments or EV transfer assays were beyond the scope of this exploratory multi-omic analysis and are planned as a necessary next step in future studies.

## 4. Materials and Methods

### 4.1. Rat Model of Allergic Asthma

Brown Norway rats (males, 7-week-old, Janvier Labs, Le Genest-Saint-Isle, France) were sensitized and challenged with house dust mite (HDM) as previously described [6,7]. Briefly, animals in the study group (*n* = 5) were subcutaneously injected with 45 µg of HDM (Citeq Biologics, Groningen, The Netherlands) in Al(OH)3 as an adjuvant once per week for three weeks and then intranasally challenged with 120 µg of HDM in saline three times per week for three weeks. Animals in the control group (*n* = 3) received saline in Al(OH)3 for subcutaneous injection for sensitization and saline intranasally for challenge. Anesthesia was not used during the HDM sensitization and challenge procedures. Rats were sacrificed by decapitation, and tissues were immediately snap-frozen in liquid nitrogen and stored at −80 °C for further analysis. Peripheral blood was collected in separator gel tubes and left for one hour at room temperature to clot. After that, the tubes were centrifuged at 1700 rcf for 10 min, and the serum was collected and stored at −80 °C. All experiments were approved by the Local Ethical Commission on Experiments on Animals at the Poznan University of Life Sciences, Poznan, Poland (agreements no. 35/2017 and 22/2023). All experimental procedures were carried out in accordance with relevant national and institutional guidelines and regulations for the care and use of laboratory animals. All methods are reported in accordance with the ARRIVE guidelines.

### 4.2. Enzyme-Linked Immunosorbent Assay (ELISA) Test

We used an ELISA to quantify IgE in rat serum according to the manufacturer’s (Elabscience Biotechnology, Houston, TX, USA) instructions. Serum samples were diluted 10 times in Reference Standard & Sample Diluent and run in duplicate. Absorbance was measured at 450 nm using an Asys UVM 340 Microplate Reader (Biogenet, Otwock, Poland).

### 4.3. Extracellular Vesicle Collection and miRNA Isolation

For extracellular vesicle isolation, we used size-exclusion chromatography (SEC) with a 70 nm qEV single column (Izon, Lyon, France). Purified fractions containing extracellular vesicles were collected using an Automatic Fraction Collector (Izon), according to the manufacturer’s instructions. Briefly, the column was flushed with 4 mL of measurement electrolyte (ME), after which 150 µL of rat serum was loaded onto the 70 nm qEV single column. The void volume (column volume) was discarded, and fractions containing extracellular vesicles (fractions 1–5) were collected, pooled, and concentrated using a Concentrator Plus (Eppendorf, Hamburg, Germany).

Fractions were lysed in QIAzol Lysis Reagent (Qiagen, Venlo, The Netherlands), and the sample volume did not exceed 10% of the lysis buffer volume. The aqueous phases were supplemented with 1 µL of RNA-grade glycogen (Thermo Fisher Scientific, Waltham, MA, USA) at a concentration of 20 µg/mL and incubated overnight at −20 °C. Following three washes with 75% ethanol, the RNA pellet was dissolved in 10 µL of nuclease-free water and stored at −80 °C for further analysis.

### 4.4. Extracellular Vesicle Characterization

Extracellular vesicle characterization was performed using tunable resistive pulse sensing (TRPS) analysis with a qNano Gold instrument (Izon). A pooled sample of fractions 1–5 from AFC isolation and the TKP200 calibrator (mode diameter: 200 nm) were diluted in 0.2 μm filtered ME by 10-fold and 500-fold, respectively. All measurements were carried out using an NP250 nanopore. Initially, NP250 nanopore was stretched to 47 mm and coated for 15 min with a wetting solution diluted in 0.2 μm filtered ME, following the manufacturer’s (Izon) protocol. The wetting solution was then removed, and the nanopore was washed with freshly prepared 0.2 μm filtered ME. At this step, a stable baseline current of 130–140 nA was established. Next, the upper fluid cell was carefully cleaned with dust-free wipes, the sample and TKP200 calibrator were loaded onto the nanopore and a pressure of 15 mbar was applied. For the measurement, the NP250 was stretched to 47.16 mm, and a voltage of 0.72 V was applied. Particle detection was performed using Classic Capture software v3.4, with RMS noise < 15 pA.

Extracellular vesicle membrane markers were characterized with Western blot. Fractions 1–5 from AFC were pooled and concentrated using the Concentrator Plus (Eppendorf). Samples were lysed in QIAzol Lysis Reagent, ensuring that the sample volume did not exceed 10% of the lysis buffer volume. Proteins were precipitated with acetone at −20 °C for 3 h. Protein pellets from three isolations were pooled, and protein concentration was measured using the Pierce BCA Protein Assay Kit (Thermo Fisher Scientific). For Western blot, 50 µg of protein was loaded onto a Novex 16% Tris-Glycine Mini Protein Gel (Thermo Fisher Scientific) and transferred onto a 0.22 µm nitrocellulose membrane using the iBlot 2 Dry Blotting System (Thermo Fisher Scientific). Membranes were blocked in 5% non-fat dry milk (NFDM) and incubated overnight with anti-CD9 (ab307085, Abcam, Cambridge, UK) and anti-CD81 (GTX135297, GeneTex, Irvine, CA, USA) antibodies. Following three washes with PBST, membranes were incubated for 1 h with goat anti-rabbit IgG secondary antibody (HAF008, Novus Biologicals, Centennial, CO, USA). The signal was developed using SuperSignal West Pico PLUS Chemiluminescent Substrate (Thermo Fisher Scientific) and imaged on the ChemiDoc Imaging System (BioRad, Hercules, CA, USA).

### 4.5. Validation of EV-Derived miRNA Expression

The TaqMan Advanced miRNA cDNA Synthesis Kit (Thermo Fisher Scientific) was used to validate EV-derived miRNA expression. The concentration of miRNA was measured using the Qubit microRNA Assay Kit (Thermo Fisher Scientific) with a Quantus Fluorometer (Promega, Madison, WI, USA). We used 1 ng of miRNA as an input for reverse transcription. We used TaqMan Advanced miRNA Assays (Thermo Fisher Scientific) to quantify the expression of EV-derived miRNAs that we previously identified as differentially expressed in lungs (rno-miR-223-3p, rno-miR-147, rno-miR-99b-5p, rno-miR-100-5p, rno-miR-186-5p, rno-let-7b-5p, and rno-miR-672-5p) and adipose tissue (rno-miR-151-5p, rno-miR-30a-5p, rno-miR-22-3p, rno-miR-146a-5p, rno-miR-127-3p, and rno-miR-21). Rno-miR-26a was used as an endogenous control. Data acquisition was done in ABI Prism 7900HT (Thermo Fisher Scientific) using SDS 2.4 software, and data analysis was performed in Data Assist v3.01 using the 2^−ΔΔCt^ method.

### 4.6. Microarray Analysis

For the expression analysis of mRNAs differentially expressed in lungs and adipose tissues, we used the SurePrint G3 Rat Gene Expression 8x60K Microarray, following the manufacturer’s (Agilent Technologies, Santa Clara, CA, USA) protocol. A total of 50 ng of RNA was used as input, and labeling was performed using the Low Input Quick Amp Labeling Kit (Agilent Technologies, Santa Clara, CA, USA). Microarray scanning was done using the SureScan Dx Microarray Scanner (Agilent Technologies). Data were analyzed in GeneSpring 15.5 GX, and an unpaired *t*-test was used for statistical analysis, with Benjamini–Hochberg correction for multiple testing and a fold-change threshold of 1.5.

### 4.7. Multi-Omic Analysis

For multi-omic analysis (MOA), we used GeneSpring 15.5 GX to analyze differentially expressed mRNAs in lungs and adipose tissue, as well as rno-let-7b-5p target genes. For target prediction, we used three databases: Target Scan release 8.0 (cumulative weighted context++ score > 0.5), miRDB (target score > 60) and microT (default score threshold of 0.7). We also included all rno-let-7b-5p validated target genes listed in miRTarBase. Only significantly altered mRNAs with an expression direction opposite to the expression of rno-let-7b-5p were included in the analysis.

### 4.8. Real-Time PCR for mRNA Quantification

RNA was extracted from lung and adipose tissue with the ExtractMe miRNA kit (Blirt, Gdansk, Poland), according to the manufacturer’s protocol. Quantitative and qualitative assessment of RNA samples was performed using a NanoDrop 2000 spectrophotometer (Thermo Fisher Scientific), with A260/280 ratios ranging between 1.8 and 2.0. A total of 500 ng of RNA was reverse-transcribed into cDNA using the GoScript Reverse Transcription System (Promega) following the manufacturer’s instructions. Real-time quantitative PCR was carried out with the GoTaq qPCR System (Promega), and gene expression levels were determined using the comparative ΔΔCt method. For qPCR, we used the following primers: *Vav3* forward 5′-AGGCAACAGCTTGCTAAGTCCC-3′ and reverse 5′-ATGGAAACCAGCCCACCCTG-3′, *Ptafr* forward 5′-CGGCTGAGCTCCTATAGGGACATAC-3′ and reverse 5′-GCAACCACGCCCAGTACAAA-3′, and Zbtb16 forward 5′-GCAGTGTGTGTGGGGTCGAA-3′ and reverse 5′-GCTTTGGCACCCGCTGAATG-3′. We used *Gapdh* and *Hprt* as endogenous controls (*Gapdh* primers: forward 5′-AACTCCCTCAAGATTGTCAGCAA-3′ and reverse 5′-GGCATGGACTGTGGTCATGA-3′; *Hprt* primers: forward 5′-TGGATACAGGCCAGACTTTGTTGG-3′ and reverse 5′-ACTTGCCGCTGTCTTTTAGGCT-3′). Data acquisition was done in ABI Prism 7900HT (Thermo Fisher Scientific) using SDS 2.4 software, and data analysis was performed in Data Assist v3.01 using the 2^−ΔΔCt^ method.

### 4.9. miRNA–RNA Interaction Analysis

To assess the interaction between rno-let-7b-5p and its target genes, we employed two independent tools: IntaRNA v.2.0 and DMISO (https://hulab.ucf.edu/research/projects/DMISO/, accessed on 1 July 2026). IntaRNA accurately predicts interactions between two RNA molecules, including eukaryotic miRNAs and their target genes [37]. It provides an energy score for each miRNA–RNA interaction, which is the sum of the hybridization free energy of the interacting RNA subsequences and the energies required to unfold the interaction sites in both molecules [38,39]. For the miRNA–target interaction, we set the minimal number of base pairs in the seed to 5. As an additional validation step, we used DMISO to analyze interaction sequences predicted by IntaRNA. DMISO is a deep learning-based tool designed for precise miRNA and isomiR target prediction and in silico validation [40]. For the DMISO computational analysis, we used binding sequences predicted by IntaRNA within the UTR region of the corresponding target genes. DMISO machine learning and computational prediction analyses were conducted using the Windows Subsystem for Linux (WSL) with Ubuntu 24.04.3 LTS as the operating system environment.

### 4.10. Statistical Analysis

All data are presented as mean ± standard deviation (SD), unless otherwise indicated. The normality of data distribution was assessed using the Shapiro–Wilk test before statistical comparisons. For comparisons between two groups, an unpaired two-tailed Student’s *t*-test was applied. For microarray data, statistical significance was assessed using an unpaired *t*-test with Benjamini–Hochberg correction for multiple testing, and a fold-change threshold of 1.5 was applied. Differential miRNA and mRNA expression levels were analyzed using the comparative ΔΔCt method. A *p*-value < 0.05 was considered statistically significant. Statistical analyses were performed using GraphPad Prism 9 (GraphPad Software, San Diego, CA, USA) and GeneSpring GX 15.5 (Agilent Technologies, Santa Clara, CA, USA). The EV-derived miRNA qPCR analysis was performed as an exploratory, targeted candidate-based analysis of 13 miRNAs selected a priori based on our previous lung and adipose tissue data. No multiple-testing correction was applied at this stage; therefore, nominal *p*-values are reported, and the rno-let-7b-5p result should be interpreted as an exploratory finding.

## 5. Conclusions

In conclusion, this preliminary and hypothesis-generating study identified a nominal 1.5-fold increase in serum EV-associated rno-let-7b-5p in HDM-exposed rats, accompanied by altered expression of its predicted targets, including increased pulmonary *Ptafr* and decreased adipose *Zbtb16*. Because the study included a small cohort and no multiple-testing correction was applied to the 13-miRNA panel, these findings should be interpreted cautiously and require confirmation in larger independent cohorts. The tissue and cellular origin of circulating EV-associated rno-let-7b-5p remains unknown, and the present data do not establish direct EV transfer or a causal mechanism of lung–adipose tissue communication. Nevertheless, the observed associations support rno-let-7b-5p as a candidate for future mechanistic studies. Functional experiments, including gain- and loss-of-function approaches and EV transfer assays, are required to validate its biological role.

## Data Availability

The datasets generated and/or analyzed during the current study are available from the corresponding author on reasonable request. The microarray datasets have been deposited in the Gene Expression Omnibus (GEO) database under accession number GSE315152 and will be released publicly upon acceptance of the manuscript.

## Supplementary Materials

The following supporting information can be downloaded at https://www.mdpi.com/article/10.3390/ijms27135989/s1.

## Abbreviations

HDM—house dust mite; EV—extracellular vesicle; miRNA—microRNA; TRPS—tunable resistive pulse sensing; SEC—size-exclusion chromatography; MOA—multi-omic analysis; PAF—platelet-activating factor; PTAFR—platelet-activating factor receptor; NLRP3—NLR family pyrin domain containing 3; GPCR—G protein-coupled receptor; ER—endoplasmic reticulum; ELISA—enzyme-linked immunosorbent assay; qPCR—quantitative polymerase chain reaction; PCA—principal component analysis; Th2—T helper type 2; NKT—natural killer T; GLP-1R—glucagon-like peptide-1 receptor.

## References

[1] Wouters E.F.M., Reynaert N.L., Dentener M.A., Vernooy J.H.J. (2009). Systemic and local inflammation in asthma and chronic obstructive pulmonary disease: Is there a connection?. Proc. Am. Thorac. Soc..

[2] Serafino-Agrusa L., Spatafora M., Scichilone N. (2015). Asthma and metabolic syndrome: Current knowledge and future perspectives. World J. Clin. Cases.

[3] Fantuzzi G. (2005). Adipose tissue, adipokines, and inflammation. J. Allergy Clin. Immunol..

[4] Forno E., Lescher R., Strunk R., Weiss S., Fuhlbrigge A., Celedón J.C. (2011). Decreased response to inhaled steroids in overweight and obese asthmatic children. J. Allergy Clin. Immunol..

[5] Thomou T., Mori M.A., Dreyfuss J.M., Konishi M., Sakaguchi M., Wolfrum C., Rao T.N., Winnay J.N., Garcia-Martin R., Grinspoon S.K. (2017). Adipose-derived circulating miRNAs regulate gene expression in other tissues. Nature.

[6] Langwinski W., Szczepankiewicz D., Narozna B., Stegmayr J., Wagner D., Alsafadi H., Lindstedt S., Stachowiak Z., Nowakowska J., Skrzypski M. (2022). Allergic inflammation in lungs and nasal epithelium of rat model is regulated by tissue-specific miRNA expression. Mol. Immunol..

[7] Szczepankiewicz D., Langwinski W., Kolodziejski P., Pruszynska-Oszmałek E., Sassek M., Nowakowska J., Chmurzyńska A., Nowak K.W., Szczepankiewicz A. (2020). Allergic Inflammation Alters microRNA Expression Profile in Adipose Tissue in the Rat. Genes.

[8] Imayama I., Eccles J.D., Ascoli C., Kudlaty E., Park G.Y. (2024). Body Weight and Allergic Asthma: A Narrative Review. J. Clin. Med..

[9] Hersoug L.G., Linneberg A. (2007). The link between the epidemics of obesity and allergic diseases: Does obesity induce decreased immune tolerance?. Allergy.

[10] Mancuso P. (2010). Obesity and lung inflammation. J. Appl. Physiol. Bethesda Md. (1985).

[11] Liu X., Gao J., Yang L., Yuan X. (2024). Roles of exosomal miRNAs in asthma: Mechanisms and applications. J. Asthma Allergy.

[12] Bartel S., La Grutta S., Cilluffo G., Perconti G., Bongiovanni A., Giallongo A., Behrends J., Kruppa J., Hermann S., Chiang D. (2020). Human airway epithelial extracellular vesicle miRNA signature is altered upon asthma development. Allergy.

[13] Soccio P., Moriondo G., Lacedonia D., Tondo P., Pescatore D., Quarato C.M.I., Carone M., Barbaro M.P.F., Scioscia G. (2023). MiRNA and exosomal miRNA as new biomarkers useful to phenotyping severe asthma. Biomolecules.

[14] Bahmer T., Krauss-Etschmann S., Buschmann D., Behrends J., Watz H., Kirsten A.M., Pedersen F., Waschki B., Fuchs O., Pfaffl M.W. (2021). RNA-seq–based profiling of extracellular vesicles in plasma reveals a potential role of miR-122-5p in asthma. Allergy.

[15] Alhamdan F., Greulich T., Daviaud C., Marsh L.M., Pedersen F., Thölken C., Pfefferle P.I., Bahmer T., Potaczek D.P., Tost J. (2023). Identification of extracellular vesicle microRNA signatures specifically linked to inflammatory and metabolic mechanisms in obesity-associated low type-2 asthma. Allergy.

[16] Taka S., Tzani-Tzanopoulou P., Wanstall H., Papadopoulos N.G. (2019). MicroRNAs in Asthma and Respiratory Infections: Identifying Common Pathways. Allergy Asthma Immunol. Res..

[17] Mandolesi G., Rizzo F.R., Balletta S., Stampanoni Bassi M., Gilio L., Guadalupi L., Nencini M., Moscatelli A., Ryan C.P., Licursi V. (2021). The microRNA let-7b-5p Is Negatively Associated with Inflammation and Disease Severity in Multiple Sclerosis. Cells.

[18] Koeppen K., Nymon A., Barnaby R., Bashor L., Li Z., Hampton T.H., Liefeld A.E., Kolling F.W., LaCroix I.S., Gerber S.A. (2021). Let-7b-5p in vesicles secreted by human airway cells reduces biofilm formation and increases antibiotic sensitivity of *P. aeruginosa*. Proc. Natl. Acad. Sci. USA.

[19] Hartung F., Haimerl P., Schindela S., Mussack V., Kirchner B., Henkel F.D.R., Bernhardt U., Zissler U.M., Santarella-Mellwig R., Pfaffl M. (2024). Extracellular vesicle miRNAs drive aberrant macrophage responses in NSAID-exacerbated respiratory disease. Allergy.

[20] Rattanapan Y., Nongwa K., Supanpong C., Satsadeedat C., Sai-ong T., Kooltheat N., Chareonsirisuthigul T. (2024). Downregulation of miR-25-3p and Its Impact on PTAFR and IGF2BP3 Expression in Type 2 Diabetes Mellitus: Implications for Biomarker Discovery and Disease Pathogenesis. J. Clin. Med. Res..

[21] Wang X., Iyer A., Lyons A.B., Körner H., Wei W. (2019). Emerging Roles for G-protein Coupled Receptors in Development and Activation of Macrophages. Front Immunol..

[22] Rosenbaum D.M., Rasmussen S.G.F., Kobilka B.K. (2009). The structure and function of G-protein-coupled receptors. Nature.

[23] Wahid H.H., Anahar F.N., Isahak N.H., Mohd Zoharodzi J., Mohammad Khoiri S.N.L., Mohamad Zainal N.H., Kamarudin N., Ismail H., Mustafa Mahmud M.I.A. (2024). Role of Platelet Activating Factor as a Mediator of Inflammatory Diseases and Preterm Delivery. Am. J. Pathol..

[24] Thawanaphong S., Nair A., Volfson E., Nair P., Mukherjee M. (2024). IL-18 biology in severe asthma. Front. Med..

[25] Hur J., Kang J.Y., Kim Y.K., Lee S.Y., Lee H.Y. (2021). Glucagon-like peptide 1 receptor (GLP-1R) agonist relieved asthmatic airway inflammation via suppression of NLRP3 inflammasome activation in obese asthma mice model. Pulm. Pharmacol. Ther..

[26] Sassmann A., Gier B., Gröne H.J., Drews G., Offermanns S., Wettschureck N. (2010). The Gq/G11-mediated signaling pathway is critical for autocrine potentiation of insulin secretion in mice. J. Clin. Investig..

[27] Harishkumar R., Hans S., Stanton J.E., Grabrucker A.M., Lordan R., Zabetakis I. (2022). Targeting the Platelet-Activating Factor Receptor (PAF-R): Antithrombotic and Anti-Atherosclerotic Nutrients. Nutrients.

[28] Olejnik A.E., Kuźnar-Kamińska B. (2024). Association of Obesity and Severe Asthma in Adults. J. Clin. Med..

[29] Kume H., Kazama K., Sato R., Sato Y. (2025). Possible Involvement of Lysophospholipids in Severe Asthma as Novel Lipid Mediators. Biomolecules.

[30] Suliman B.A., Xu D., Williams B.R.G. (2012). The Promyelocytic Leukemia Zinc Finger Protein: Two Decades of Molecular Oncology. Front. Oncol..

[31] Lopez-Castejon G. (2020). Control of the inflammasome by the ubiquitin system. FEBS J..

[32] Stutz A., Kolbe C.C., Stahl R., Horvath G.L., Franklin B.S., van Ray O., Brinkschulte R., Geyer M., Meissner F., Latz E. (2017). NLRP3 inflammasome assembly is regulated by phosphorylation of the pyrin domain. J. Exp. Med..

[33] Dong D., Du Y., Fei X., Yang H., Li X., Yang X., Ma J., Huang S., Ma Z., Zheng J. (2023). Inflammasome activity is controlled by ZBTB16-dependent SUMOylation of ASC. Nat. Commun..

[34] Pobezinsky L.A., Etzensperger R., Jeurling S., Alag A., Kadakia T., McCaughtry T.M., Kimura M.Y., Sharrow S.O., Guinter T.I., Feigenbaum L. (2015). Let-7 miRNAs target the lineage-specific transcription factor PLZF to regulate terminal NKT cell differentiation and effector function. Nat. Immunol..

[35] Zúñiga L.A., Shen W.J., Joyce-Shaikh B., Pyatnova E.A., Richards A.G., Thom C., Andrade S.M., Cua D.J., Kraemer F.B., Butcher E.C. (2010). IL-17 Regulates Adipogenesis, Glucose Homeostasis, and Obesity. J. Immunol..

[36] Huang Z., Zhao X., Wen W., Shi R., Liang G. (2025). Exosome miRNA sorting controlled by RNA-binding protein-motif interactions. Extracell. Vesicles Circ. Nucleic Acids.

[37] Mann M., Wright P.R., Backofen R. (2017). IntaRNA 2.0: Enhanced and customizable prediction of RNA-RNA interactions. Nucleic Acids Res..

[38] Busch A., Richter A.S., Backofen R. (2008). IntaRNA: Efficient prediction of bacterial sRNA targets incorporating target site accessibility and seed regions. Bioinformatics.

[39] Wright P.R., Georg J., Mann M., Sorescu D.A., Richter A.S., Lott S., Kleinkauf R., Hess W.R., Backofen R. (2014). CopraRNA and IntaRNA: Predicting small RNA targets, networks and interaction domains. Nucleic Acids Res..

[40] Talukder A., Zhang W., Li X., Hu H. (2022). A deep learning method for miRNA/isomiR target detection. Sci. Rep..

