# Supplementary figures and images for: Serum EV-Derived rno-let-7b-5p Is Associated with Lung–Adipose Alterations in Allergic Asthma
Source: Int J Mol Sci. 2026 Jul 3;27(13):5989. doi: 10.3390/ijms27135989 (PMC13362484; doi:10.3390/ijms27135989)

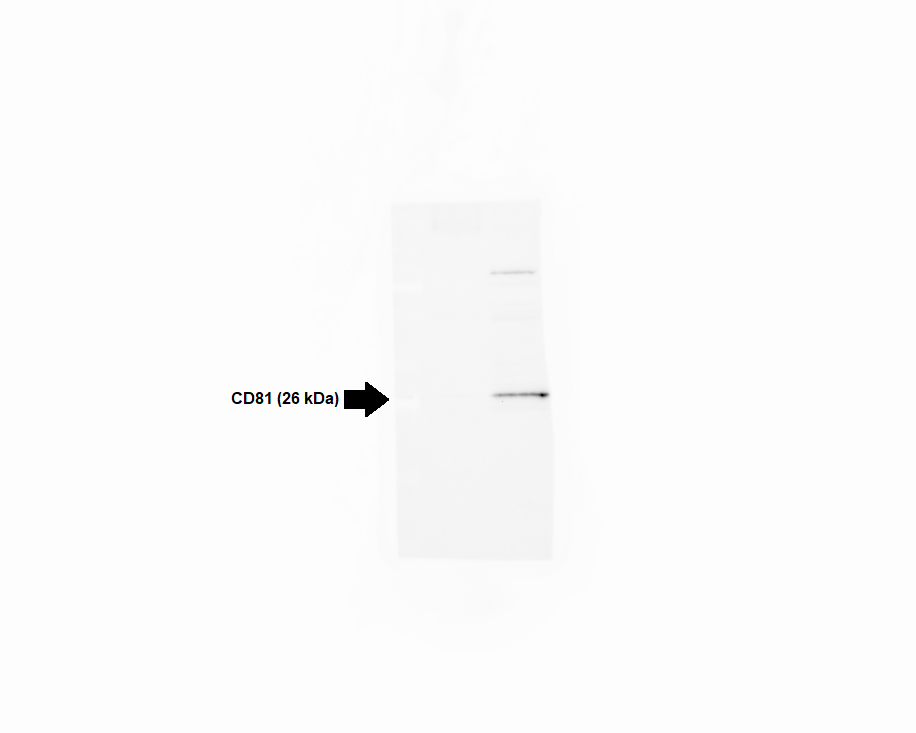

Supplement: Supplementary file 1 [file ijms-27-05989-s001.zip › Supplementary Figure S1.tif]

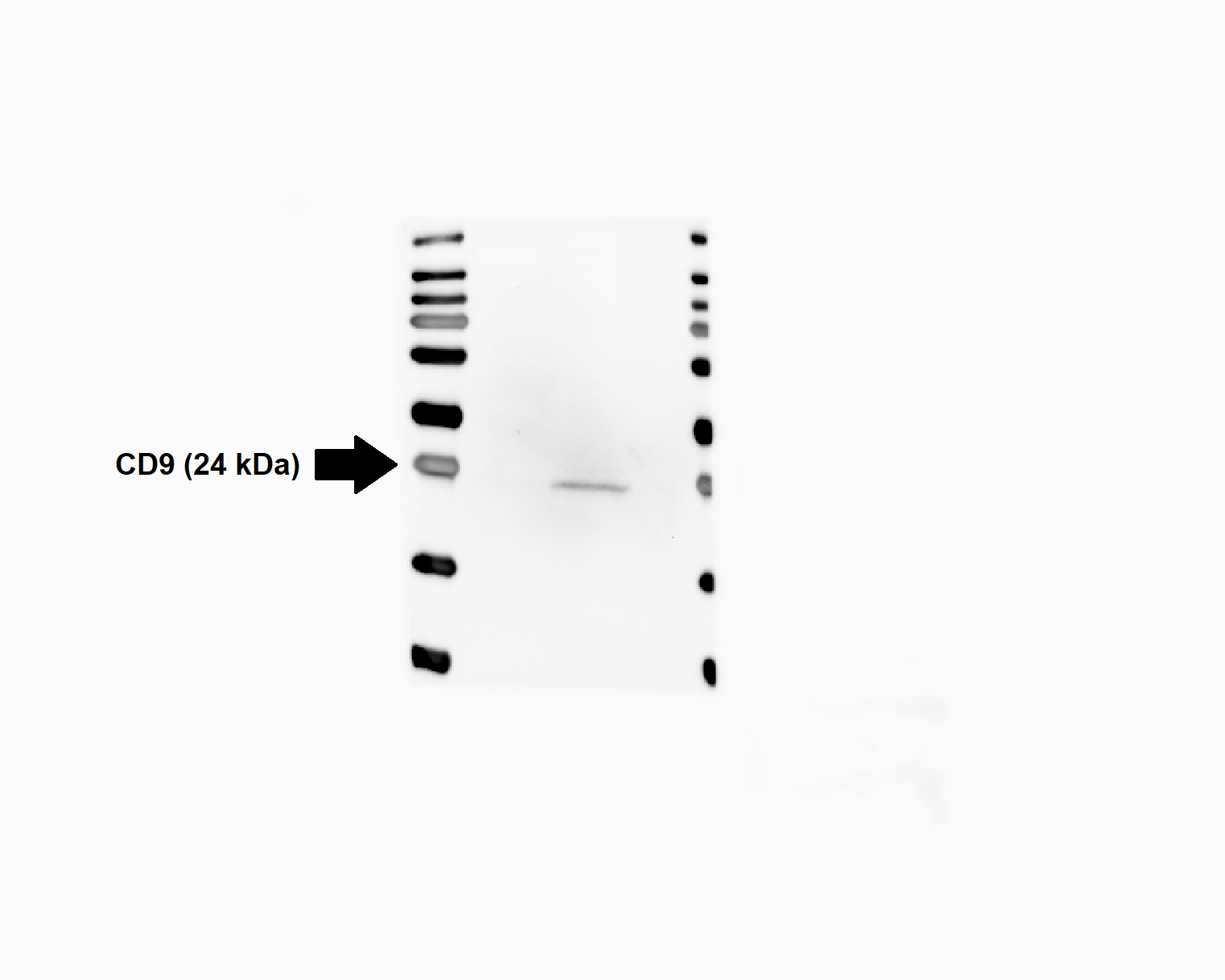

Supplement: Supplementary file 1 [file ijms-27-05989-s001.zip › Supplementary Figure S2.tif]

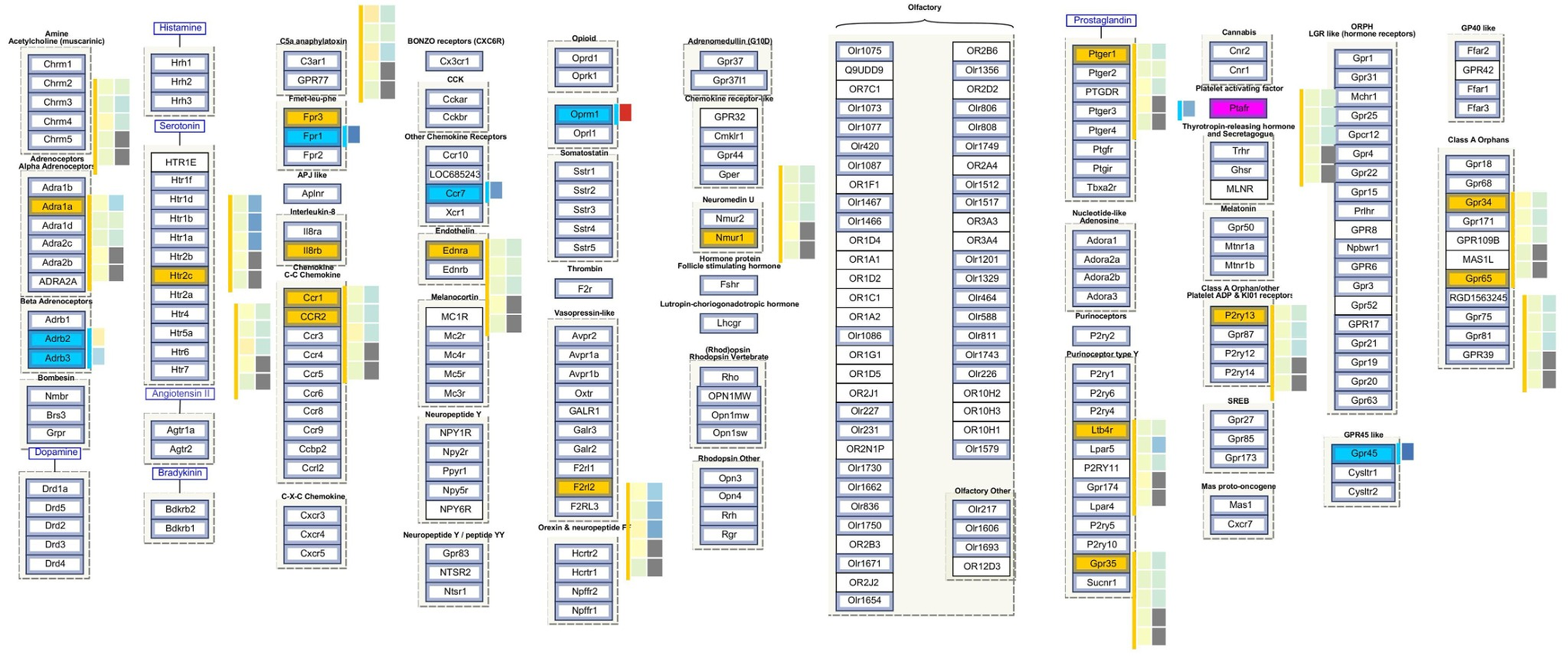

Supplement: Supplementary file 1 [file ijms-27-05989-s001.zip › Supplementary Figure S3.tiff]

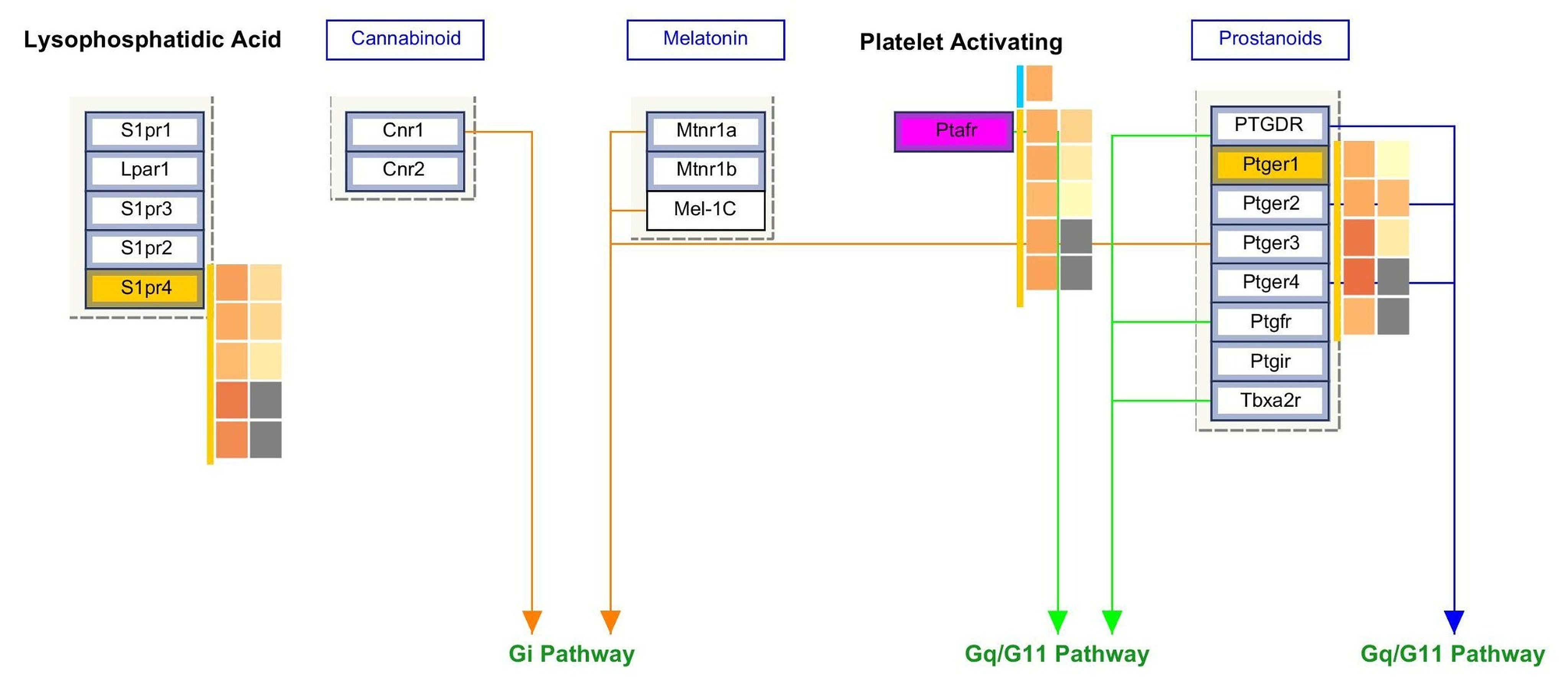

Supplement: Supplementary file 1 [file ijms-27-05989-s001.zip › Supplementary Figure S4.tiff]

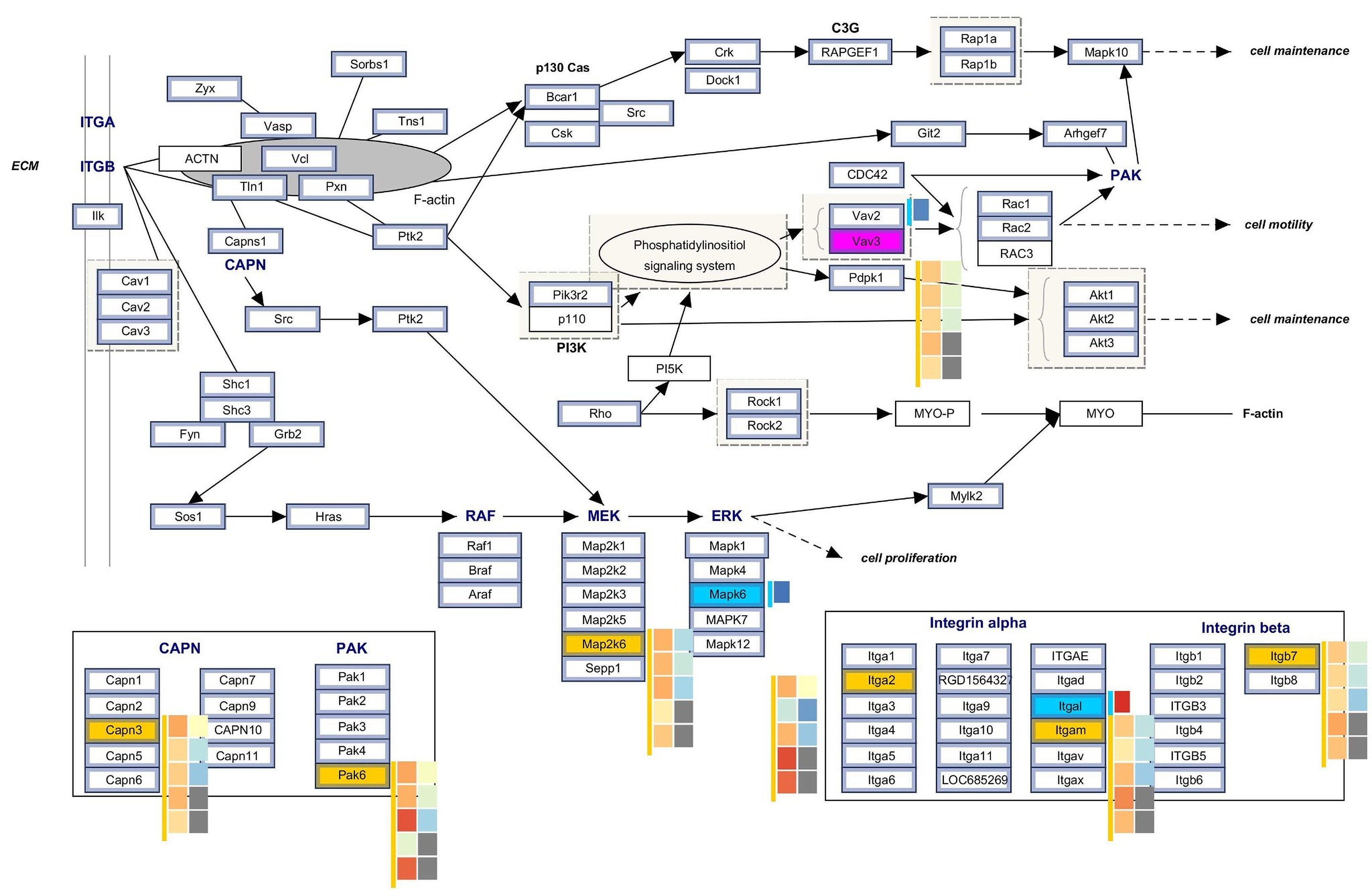

Supplement: Supplementary file 1 [file ijms-27-05989-s001.zip › Supplementary Figure S5.tiff]

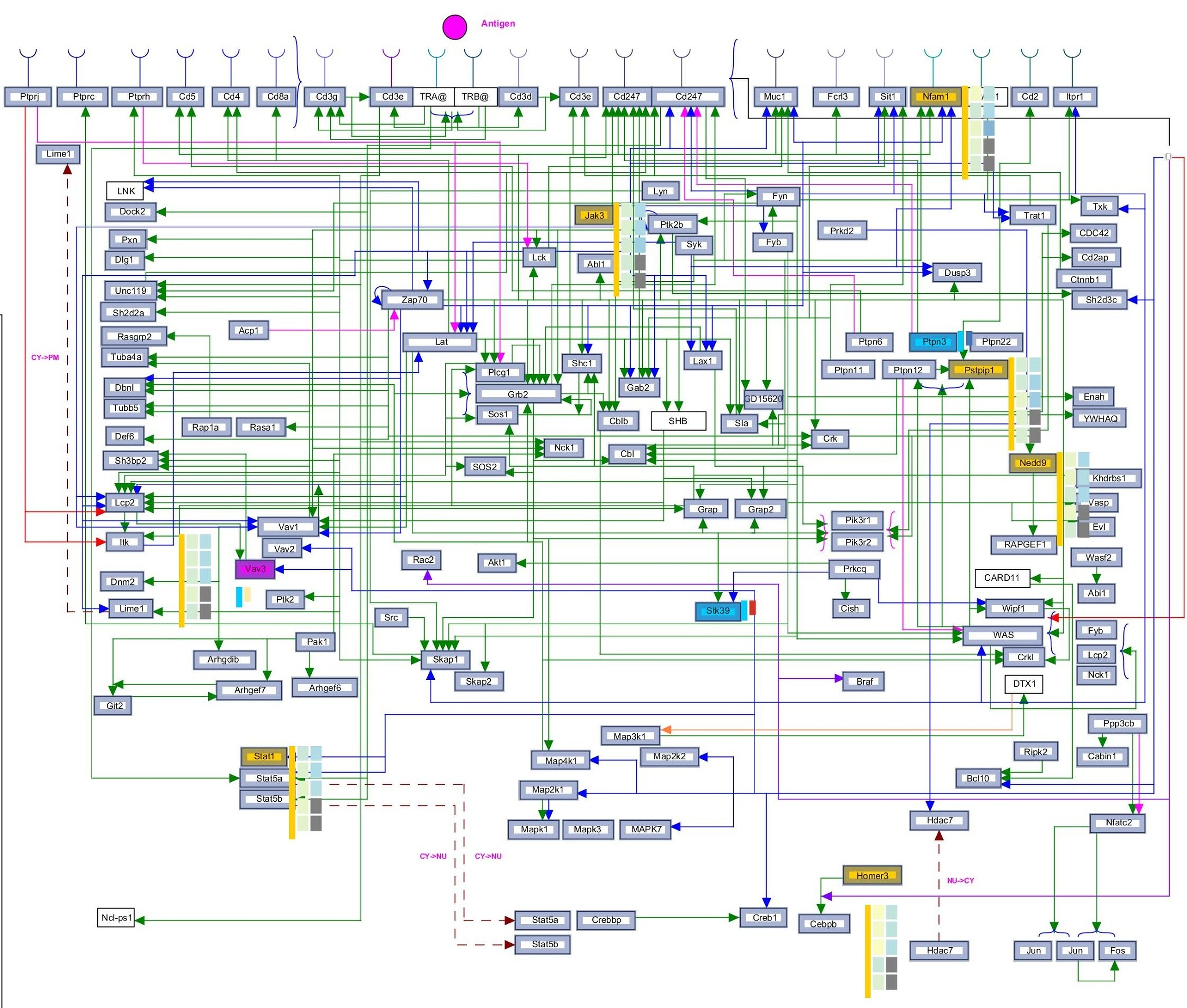

Supplement: Supplementary file 1 [file ijms-27-05989-s001.zip › Supplementary Figure S6.tiff]
